# Development, validation and evaluation of an analytical method for the determination of monomeric and oligomeric procyanidins in apple extracts

**DOI:** 10.1016/j.chroma.2017.03.030

**Published:** 2017-04-28

**Authors:** Wendy J. Hollands, Stefan Voorspoels, Griet Jacobs, Kjersti Aaby, Ane Meisland, Rocio Garcia-Villalba, Francisco Tomas-Barberan, Mariusz K. Piskula, Deborah Mawson, Irena Vovk, Paul W. Needs, Paul A. Kroon

**Affiliations:** a Institute of Food Research, Norwich Research Park, Norwich, UK; b VITONV, Flemish Institute for Technological Research, Boeretang 200, 2400 Mol, Belgium; c Nofima, Norwegian Institute of Food, Fisheries and Aquaculture Research, Ås, Norway; d Research Group on Quality, Safety and Bioactivity of Plant Foods, CEBAS-CSIC, Campus de Espinardo, Murcia, Spain; e Institute of Animal Reproduction and Food Research, Polish Academy of Sciences, Olsztyn, Poland; f LGC, Fordham, Cambridge, UK; g National Institute of Chemistry, Ljubljana, Slovenia

**Keywords:** Flavanols flavan-3-ols, Tannins, Polyphenols, Phenolics, HPLC, Inter-laboratory evaluation

## Abstract

There is a lack of data for individual oligomeric procyanidins in apples and apple extracts. Our aim was to develop, validate and evaluate an analytical method for the separation, identification and quantification of monomeric and oligomeric flavanols in apple extracts. To achieve this, we prepared two types of flavanol extracts from freeze-dried apples; one was an epicatechin-rich extract containing ∼30% (w/w) monomeric (−)-epicatechin which also contained oligomeric procyanidins (Extract A), the second was an oligomeric procyanidin-rich extract depleted of epicatechin (Extract B). The parameters considered for method optimisation were HPLC columns and conditions, sample heating, mass of extract and dilution volumes. The performance characteristics considered for method validation included standard linearity, method sensitivity, precision and trueness. Eight laboratories participated in the method evaluation. Chromatographic separation of the analytes was best achieved utilizing a Hilic column with a binary mobile phase consisting of acidic acetonitrile and acidic aqueous methanol. The final method showed linearity for epicatechin in the range 5–100 μg/mL with a correlation co-efficient >0.999. Intra-day and inter-day precision of the analytes ranged from 2 to 6% and 2 to 13% respectively. Up to dp3, trueness of the method was >95% but decreased with increasing dp. Within laboratory precision showed RSD values <5 and 10% for monomers and oligomers, respectively. Between laboratory precision was 4 and 15% (Extract A) and 7 and 30% (Extract B) for monomers and oligomers, respectively. An analytical method for the separation, identification and quantification of procyanidins in an apple extract was developed, validated and assessed. The results of the inter-laboratory evaluation indicate that the method is reliable and reproducible.

## Highlights

• Method for simultaneous determination of individual apple procyanidins and catechins is presented.
• Procyanidins separated on a HILIC column.
• Accurate quantification achieved using isolated procyanidin oligomers.
• Method validated via an inter-laboratory evaluation.

## 1 Introduction

**Fig. 1:**
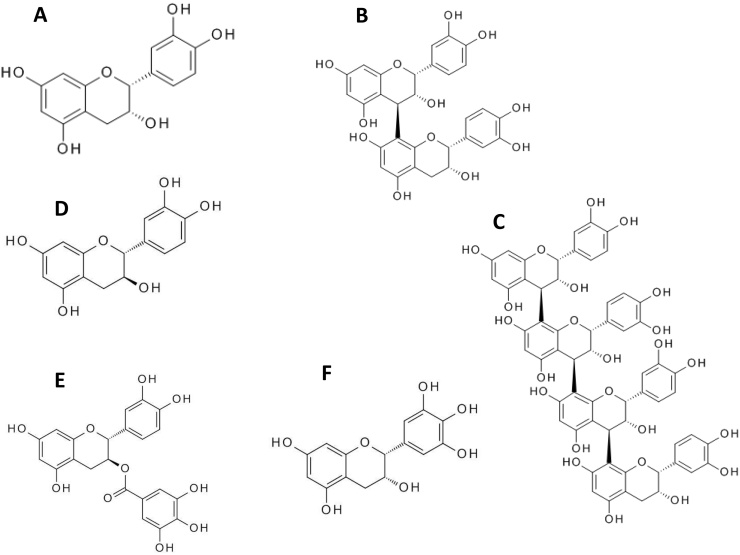
Structures of monomeric and oligomeric flavan-3-ols. A, (−)‐epicatechin (flavan-3-ol monomer); B, a procyanidin dimer (dp2); C, a tetrameric procyanidin (dp4); D, (+)‐catechin (monomer); E, (+)-catechin gallate; F, (+)‐epigallocatechin. The dimeric procyanidin shown is procyanidin B2 (an epicatechin dimer linked 8-4).

Flavanols are a sub-class of flavonoids composed of monomeric ‘catechins’ (e.g. catechin, epicatechin, epigallocatechin) and oligomeric/polymeric proanthocyanidins which are comprised of two or more catechin subunits (Fig. 1). Flavanols are potentially important dietary components because cardiovascular health benefits have been reported in numerous human intervention trials after ingestion of flavanol-rich foods and beverages [1], [2], [3], [4]. Underpinning the establishment of a causal effect for the health benefits observed in these trials is the development of validated methods for the accurate and precise quantification of the monomeric and oligomeric flavanols present in these foods. Indeed, whether the beneficial effects observed in humans caused by supplementing diets with cocoa or apple polyphenol extracts are due to the monomeric flavan-3-ols (epicatechin, catechin) or the oligomeric procyanidins is an ongoing debate. On the one hand, the data supporting (−)-epicatechin as the major cause of improvements in flow mediated dilatation (FMD) and blood pressure after cocoa consumption are mixed [5], [6] whereas the responses to cocoa that contains both epicatechin and procyanidin oligomers is stronger and more consistent [7]. The authors are not aware of any studies that have assessed the effects of a procyanidin-only (i.e. monomer-free) dietary interventions on FMD or blood pressure. Procyanidin oligomers have been shown to have potent biological activity in vitro [8], [9], but their bioavailability has been shown to be extremely limited with only dimers and occasionally trimers reported to have reached human plasma in measurable but very low concentrations. The report by Garcia-Conesa et al. [8] is particularly interesting because they show that whereas treatment of human umbilical vein endothelial cells (HUVEC) with a mixture of procyanidin oligomers of average size 4 units (8.9 μM) caused significant changes in the expression of >1000 genes, the treatments with (−)-epicatechin (25 μM) of procyanidin dp2 caused the expression of only a few genes to be altered significantly and these were considered to largely be accounted for by the false discovery rate. Since there is evidence that both the monomeric and oligomeric flavan-3-ols have biological activity, and the contribution of each type is not clear, it is important to quantify individual flavan-3-ol oligomers in addition to the monomers in foods and extracts used in dietary interventions.

A number of approaches have been used for quantifying and determining the structural nature of flavanols. Monomeric flavanols are routinely quantified with widely used techniques such as HPLC with UV, fluorescence, mass spectrometry or coulometric array detection. The analysis of oligomeric flavanols are far more challenging for a number of reasons including the potential for large molecular weight range of oligomers through polymers that may be present in single samples and each oligomer with a particular degree of polymerisation (dp) being comprised of multiple isomeric structures [10]. Further, the increasingly poor sensitivity of most detector types as the dp of the proanthocyanidins increases, and difficulties in separating the individual isomers, especially as the dp increases, offer additional challenges. Methods that seek to label the terminal catechin unit of each proanthocyanidin prior to hydrolysing the oligomers/polymers into single units are suitable for the accurate quantification of total flavanols in a sample and provide an estimate of the average dp [11], [12]. However, they do not provide information of the concentrations of individual procyanidin oligomers. More recently, chromatographic methods that seek to separate the individual dp fractions have been established and come into wider use. For example, the USDA have published food composition tables for procyanidins in foods [13] on the basis of a silica column-based separation of proanthocyanidins aqueous alcohol extracts [14]. More recently, a method for the accurate quantification of individual cocoa procyanidins up to dp10 was reported, and this was based on separation of the individual oligomers using a bonded diol stationary phase-based HPLC column. Importantly, accurate quantification of procyanidins from dp2 through 10 was achieved by isolating each of the oligomers and using these as individual reference standards [15]. The Robbins et al. report [15] highlights an important challenge in the analysis of oligomeric procyanidins, that is the lack of commercially available analytical reference standards, with the exception of a few dimers (e.g. A2, B1 and B2) and trimers (e.g. C1). The fact that each plant food containing proanthocyanidins will contain different types of ‘catechins’ as oligomeric units and different ratios of isomeric structures within a single dp necessitates the isolation of proanthocyanidin oligomers for use as reference standards for each plant food product, in order to ensure relevant relative response factors (RRFs) are established for the different oligomeric homologues. Once such RRFs are established and validated, epicatechin calibration would suffice to assess the oligomeric procyanidin content.

There are multiple reports describing the content of flavanol monomers (−)-epicatechin, (+)-catechin and procyanidin dimers and occasionally trimers in apple extracts [16], [17] and reports providing average dp values for procyanidins from apples [12]. However, apart from the USDA database which provide values for monomers, dimers, trimers, 4–6mers, 7–10mers and polymers [13], we are not aware of any reports providing accurate quantification of individual procyanidin oligomers for apples/apple products.

The objectives of this study were: (i) to develop a robust and reliable analytical method for the extraction, separation and identification of procyanidins in apple extracts; (ii) to determine RRF between procyanidin oligomers and epicatechin for the purpose of accurate quantification using procyanidins isolated in-house from an apple extract; (iii) to validate the obtained method and estimate the associated measurement uncertainty; and (iv) to perform an inter-laboratory assessment exercise to determine reproducibility of the established methodology.

## 2 Materials and methods

### 2.1 Chemicals/reagents

Epicatechin, dimethylsulfoxide (DMSO), and acetic acid were purchased from Sigma-Aldrich (Poole, UK). HPLC grade methanol, acetonitrile and hexane were purchased from Fisher Scientific (Loughborough, UK). Analytical standards dp2-10 were synthesised in-house from an apple extract and the procedure is described in Section 2.3.

### 2.2 Production of the apple extracts

The apple extracts were provided by Coressence Ltd. Extracts were produced through supercritical fluid extraction of apples that had been freeze-dried and then processed to prepare i) an epicatechin-rich extract containing around 30% (w/w) of monomeric (−)-epicatechin whilst retaining oligomeric procyanidins (Extract A) and ii) an oligomeric procyanidin rich extract that was depleted of epicatechin (Extract B).

### 2.3 Isolation of individual oligomers from an apple extract by preparative HPLC

Pre-purification of the apple extract was performed as follows: MN Polyamide (28 g) was packed into a Biotage cartridge (15 × 3.7 cm; id single fit reservoir) to a depth of 9 cm. Apple extract (4 g) was dissolved in methanol (50 mL) and mixed with polyamide (6 g). The mixture was evaporated to dryness and loaded onto the pre-wetted polyamide column. The column was connected to a Gilson preparative system comprising two 306 pumps, an 806 manometric module, an 816b dynamic mixer and a UV detector. The column was eluted isocratically at 45 mL/min with water, acetonitrile/water (30:70; *v/v*) and acetone/water (75:25; *v/v*). Fractions were monitored at 290 nm. The acetone/water fraction was then evaporated (50 °C) under reduced pressure to give a mixture of procyanidins.

Preparative HPLC was performed using an Agilent system (HP1260), equipped with two infinity preparative pumps, a dual loop autosampler, a diode array detector and a fraction collector. Samples were injected from high recovery vials (screw thread; 5 mL; Agilent) and fractions collected into tubes (14 mL; 16 × 100 mm). Both the injector and collector were cooled to 4 °C. Apple extract (100–200 mg) was dissolved in methanol (1 mL) and loaded on to a semi-preparative column (Luna HILIC 250 × 21.2 mm; 5 μm), which was eluted at 10 mL/min. Samples were eluted with acetonitrile (A) and 97% methanol and 3% water (B) as follows: 0–3 min, isocratic 7% B; 3–12 min, linear gradient 7–10% B; 12–20 min, linear gradient 10–22% B; 20–70 min, linear gradient 22–65% B; 70–80 min, linear gradient 65–100 B; 80–85 min; linear gradient 100–7% B; 85–90 min, isocratic 7% B. Post column, the eluent passed through a fluorescence detector using wavelengths 230 nm for excitation and 321 nm for emission and a diode array detector using wavelength 280 nm. Appropriate fractions were pooled and evaporated at 50 °C under reduced pressure.

### 2.4 Removal of lipid fraction from NIST SRM 2384

The NIST reference baking chocolate was defatted prior to analysis. Removal of the lipid fraction involved grinding a portion of the NIST chocolate bar into a powder using a household coffee grinder. The chocolate powder was then weighed into a pre-weighed conical flask before the addition of hexane. Samples were mixed thoroughly and then allowed to settle. The hexane layer was subsequently decanted. The process was repeated three more times, filtering and combining the hexane fractions each time. Residual hexane was evaporated from the chocolate powder to complete dryness in an air oven at 60 °C. At the end of the process, the weight of defatted chocolate was recorded. The combined hexane extracts were evaporated to complete dryness. The remaining fat was weighed. The mass balance between the extracted fat and defatted chocolate was within the expected uncertainty (less than 1.1%). Based on the removed fat fraction, reference values for epicatechin were adjusted accordingly.

### 2.5 Preparation of analytical standards

Epicatechin powder was dried in an air oven (60 °C; 2 h) and then cooled at room temperature in a desiccator. The powder was weighed (0.04 g) into a volumetric flask (10 mL) and filled to volume with DMSO to yield a 4 mg/mL stock solution. Prior to analysis of the extracts, the stock solution of standard was further diluted with 70% methanol over the range 5–100 μg/mL such that the working concentrations contain the same quantity of DMSO.

### 2.6 Method optimisation

Our finalized method for the extraction and chromatographic separation of monomeric and oligomeric procyanidins from an apple extract is described further on in Section 2.7. The parameters that were optimised are described below.

#### 2.6.1 Alternative HPLC columns and conditions

Apple extracts were analysed by HPLC (Agilent HP1100) equipped with a quaternary pump, cooled autosampler, column oven and photodiode and fluorescence detectors. The columns and HPLC conditions tested were:

i) Luna Silica column (Phenomenex; 250 × 4.6 mm; 5 μm) and a mobile phase consisting of dichloromethane (A), methanol (B) 50% acetic acid (C). Samples were eluted with an increasing gradient of (B), 0 min, 14%; 30 min, 28.4%; 45 min, 39.2%; 50 min, 86% at a flow rate of 1 mL/min. Fluorescence detection was achieved using wavelengths 276 nm for excitation and 316 nm for emission.
ii) Develosil diol column (Phenomenex; 250 × 2 mm; 5 μm) and a binary mobile phase consisting of 98% acetonitrile and 2% acetic acid (A) and 95% methanol, 3% water and 2% acetic acid (B). Samples were eluted with an increasing gradient of (B), 0 min, 7%; 3 min, 7%; 15 min, 23%; 70 min, 65%; 85 min, 100% at a flow rate of 0.5 mL/min. Fluorescence detection was achieved using wavelengths 230 nm for excitation and 321 nm for emission.
iii) Luna Hilic column (Phenomenex; 250 × 4.6 mm; 5 μm) and HPLC conditions as described for (ii) above.
iv) Luna Hilic column (Phenomenex 150 × 2.0 mm; 3 μm) with the HPLC conditions described in Section 2.7.

#### 2.6.2 Optimisation of extraction

Extraction speed can be temperature dependant. To test the effects of increasing temperature on extraction efficiency, aliquots of apple extract (40 mg; n = 3) were extracted with 70% methanol at room temperature and at 60 °C.

To assess whether filtration has an effect on the analytical process, an epicatechin standard solution was prepared at two different concentrations and applied to the HPLC column with and without filtration.

#### 2.6.3 Sample mass and dilution

To assess the effect of sample mass on monomeric and oligomeric procyanidin conc., apple extract (20, 40 and 100 mg; n = 3/mass) were dissolved in 70% methanol such that the final dilution volume remained the same (100 mL).

To assess the effects of the dilution procedure, two dilution methods were investigated. Method A; Apple extract (∼100 mg; n = 3) was weighed into a volumetric flask (25 mL) and filled to volume with 70% methanol. The extract was then diluted a further 2, 4 and 8-fold. The final dilution volumes were 25 (original stock solution), 50, 100 and 200 mL. Method B; Apple extract (∼100 mg) was weighed directly into 25, 50, 100 and 200 mL volumetric flasks (n = 3/conc.) and filled to volume with 70% methanol. No further dilution was applied. For both methods, extracts were processed as described in Section 2.7 and then sub-samples of each concentration of extract centrifuged before applying to HPLC.

### 2.7 Optimized method for the analysis of the apple extract

The final in-house method developed for the extraction of monomeric and oligomeric procyanidins from the apple extract involved the weighing of a solid sample (∼100 mg) into a volumetric flask (50 mL) before partially filling with pre-warmed (60 ^°^C) aqueous methanol (70%). The dissolution was sonicated in a water bath (60 ^°^C; 10 min) to disperse the sample before incubating for 30 min at 60 ^°^C. Post incubation, flasks were cooled and then filled to volume with 70% aqueous methanol. Sub-samples of the dissolution were centrifuged (2500*g*; 5 min) before applying to HPLC. Apple extract A (epicatechin rich extract) was diluted a further 10-fold to avoid a saturation effect of epicatechin on the HPLC florescence detector and therefore ensure it remained within the HPLC detection limits. Apple extracts were prepared concurrently with a certified reference material (NIST SRM 2384–baking chocolate; Section 2.4) with a known epicatechin conc. as a measure of analytical accuracy.

For chromatographic separation of the monomers and oligomers, extracts were analysed by HPLC (Agilent HP1100) equipped with a quaternary pump, cooled autosampler, column oven and photodiode and fluorescence detectors. The final selected column was a Luna Hilic column (150 × 2.0 mm; 3 μm) (Phenomenex). The mobile phase consisted of 98% acetonitrile and 2% acetic acid (A) and 95% methanol, 3% water and 2% acetic acid (B). Samples were eluted with an increasing gradient of (B): 0 min, 7%; 3 min, 7%; 15 min, 30%; 40 min, 49%; 40.1 min, 7% and 45 min 7% at a flow rate of 0.350 mL/min. The total run time was 45 min, the mid-peak RT of dp 10 being 38 min. The injection volume was 2 μL. The column temperature was held at 35 °C. The fluorescence detector wavelengths were 230 nm for excitation and 321 nm for emission. To ensure that all analytes were within the linear range of the detector, the photomultiplier tube gain (PMT) was set at 9. Monomeric content of the extracts was calculated relative to a response factor derived from an authentic epicatechin standard curve over the range 0–100 μg/mL. Oligomeric procyanidins (dp2-10) were calculated against the response factor obtained for epicatechin and additional relative fluorescence response data determined from procyanidins isolated in-house (Section 2.3) for use as analytical standards.

### 2.8 Method validation

The performance characteristics considered for validation of the optimized method were: selectivity, linearity, working range, RRFs, limit of quantification (LOQ), precision, trueness and method uncertainty. A procyanidin rich apple extract was used for the experiments.

#### 2.8.1 Selectivity

A high degree of selectivity was achieved by using fluorescence detection with excitation and emission wavelengths that are specific for flavan-3-ols [18]. As chromatographic resolution is insufficient to separate individual compounds within each dp-class, the response per dp-class was summed. No further assessment of specificity was done.

#### 2.8.2 Linearity and working range

Linearity of the epicatechin calibration was assessed visually and by means of a lack-of-fit test [19].

The working range was defined as the interval between the upper and the lower levels of the analyte within the calibration curve.

#### 2.8.3 Determination of RRFs

Authentic reference standards for apple oligomeric procyanidins are not commercially available. During the method development and validation, RRFs for dp2-10 vs epicatechin were established. To this extent the pure dp’s that were isolated in-house were used. RRFs were determined in triplicate and at two independent laboratories. The RRFs were incorporated into the final analytical method for quantification of procyanidins and was further validated through an inter-laboratory evaluation exercise. Calibration curves for epicatechin and each oligomer (dp2-10) were measured over the conc. range 5–100 μg/mL (n = 3/conc.) on three different days.

#### 2.8.4 Limit of quantification (LOQ)

The method was applied to samples with relatively high epicatechin and procyanidin levels, therefore practical tests on LOQ were not performed. When the method calibration could sensibly be forced through zero, LOQ was determined by system performance. In other cases, theoretical estimates of the LOQ based on calibration curves were obtained using the following formula:

(1) $$LOQ=\frac{10\sigma }{S}$$

where σ is the standard deviation of the response and S is the slope of the calibration curve

#### 2.8.5 Method precision

To determine the intra-day (repeatability) and inter-day (intermediate) precision of the methodology, replicate samples of apple extract (n = 3) were analysed three times on the same day and over three separate days. One-way ANOVA was used to calculate repeatability and intermediate precision.

Reproducibility of the RRF-determination was assessed separately. To this extent, the RRFs for the individual oligomers were determined at two independent locations, by different analysts, using different instrumentation. Variation of the RRFs can be used for reproducibility assessment. Variation within the RRFs of dp2-10 vs epicatechin adds to the method uncertainty and was considered in the final uncertainty estimation.

Reproducibility of the entire method was assessed in an inter-laboratory exercise. Eight laboratories in eight countries across Europe participated in the evaluation of the method for the quantification of procyanidins in an apple extract. Each laboratory received the following: 1) the method protocol describing the preparation of the calibration standards, apple extracts and control sample; the required HPLC conditions and sample sequence set up; example chromatograms and guidance on peak integration procedure; guidance on calculation of the procyanidin content of the test materials using a commercially available (−)-epicatechin standard and RRFs for dp 2–10 as established earlier 2) an excel calculation spreadsheet and reporting template and 3) two different apple extracts labelled A and B, and a control sample (defatted chocolate). Each participant conducted six independent measurements of the two apple extracts over two different days (n = 3 replicates/extract/day) and two independent measurements of the control sample (n = 1 replicate/day).

#### 2.8.6 Method trueness

The determination of trueness (the closeness of agreement between the average value obtained from a set of test results and an accepted reference/“true” value) can only be established by means of a certified reference material (CRM). However, since no CRM was available, the recovery of analytes in fortified samples was investigated.

A spiking experiment was set up for the estimation and evaluation of the recovery. The extract was fortified with procyanidins to obtain fortified samples containing approximately 100% and 200% of the expected content. Three replicates per spiking level were prepared and analysed, as well as three replicates of an unfortified sample. The mean analyte concentrations obtained for the spiked samples (in μg/mg) were referred to the amount added to the samples in order to find an estimate of the response originated by the analytes spiked in the samples. The expected values were plotted against the concentrations that were actually found.

#### 2.8.7 Stability of the extracts

Epicatechin and its oligomers are compounds well known to be stable in fruit juices [20] and during gastro-intestinal transit [21]. Therefore no extensive stability testing was executed during method validation. To test the stability of the analytes in the apple extracts, a small stability study was performed. Samples were measured at 0 h and after 48, 96 and 168 h.

#### 2.8.8 Method uncertainty

The maximum expanded uncertainty (U) is based on the combined uncertainty resulting from the different uncertainty contributions (u) throughout the method. In this case U was estimated taking into account the different contributions specified in the following expression:

(2) $$U=k*\sqrt{u_{rep}{\left(RRF\right)}^{2}+u_{il}{\left(RRF\right)}^{2}+\frac{u_{r}{}^{2}}{n_{1}}+\frac{u_{ip}{}^{2}}{n_{2}}+u_{bias}^{2}+u_{rec}^{2}}$$

Where:

U – expanded uncertainty;

k – coverage factor (k = 2) resulting in a confidence level of approximately 95%;

u_rep_ (RRF) – uncertainty resulting from repeatability of RRF within lab,

u_il_ (RRF) – uncertainty resulting from variation of RRF between labs,

u_r_ – uncertainty resulting from repeatability,

u_ip_ – uncertainty from the intermediate precision,

n_1_ – total number of analyses,

n_2_ – number of days measured,

u_bias_ – u resulting from the measurement bias,

u_rec_ – u resulting from the recovery.

## 3 Results and discussion

### 3.1 Method optimisation

#### 3.1.1 Alternative HPLC columns and conditions

Optimization of the chromatographic separation of the oligomers was based on degree of polymerisation (i.e. molecular weight) and employing several HPLC columns and appropriate mobile phase techniques. Analyte peak identification was based upon retention time match with the reference standards (dp2-10) isolated in-house combined with excitation and emission wavelengths.

**Fig. 2:**
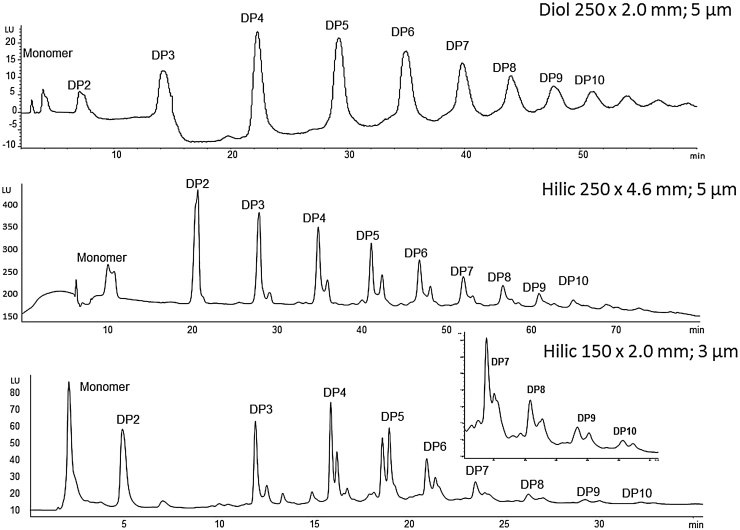
Comparison between columns in the chromatographic separation of monomeric and oligomeric procyanidins in an apple extract.

Initially, our chromatographic separation of procyanidins used a normal phase silica column. However, because of the complexity in the structural diversity of oligomers in the apple extract, this method was not suitable for quantification purposes, particularly at the higher dp. Thus, we modified our chromatographic method to use a diol column as this was reported to give improved detection of oligomers at the higher dp, whilst avoiding fluorescence response suppression issues inherent in the use of chlorinated solvents. The method was adapted from that of Robbins et al. [15] and is described in Section 2.6.1. Whilst chromatographic resolution of each dp peak was achieved, the structural isomers within each dp were not apparent. It was expected that a HILIC column might give even better resolution than the Diol column with the same eluent mixture. Therefore, we compared the Develosil diol column with the Luna HILIC column, which confirmed the hypothesis of HILIC superiority for this application. The Luna Hilic column bears cross-linked diol groups, which adds both functionality and robustness to the column compared to the Develosil diol column. Substructure, attributable to a mixture of structural isomers, was now apparent in each dp peak and resolution of the higher dp’s was also improved. However, a drawback of these column dimensions was the lengthy 90 min chromatographic run time. To increase sample throughput we investigated an alternative HILIC column, with modified HPLC conditions to reduce the run time to 45 min. Fig. 2 shows the differences in separation and resolution of the monomeric and oligomeric procyanidins between the diol and two HILIC columns. One of the limitations of our final chromatographic method is the lack of separation of the monomeric catechins ((−)-epicatechin and (+)-catechin) in the apple extract.

#### 3.1.2 Influence of extraction at elevated temperature and filtration

The monomeric and oligomeric conc. of the apple extract was higher in the heated samples (60 ^°^C) compared with the non-heated samples (17 and 13%; p = 0.01 and 0.14; monomers and oligomers respectively) indicating that heating apple extracts may improve the extraction efficiency.

For both concentrations tested, filtering samples resulted in an increase in epicatechin conc. (>5.5%). Since samples of apple extract were routinely centrifuged prior to filtering, and was sufficient to achieve sample clarification, the filtration step was not retained.

#### 3.1.3 Sample mass and dilution

As expected, the largest sample mass (100 mg) resulted in a lower co-efficient of variation between replicates in the procyanidin conc. of the apple extract (Table 1). Whilst the variation between replicates was acceptable at 40 mg of extract, we chose to incorporate the larger sample mass into our final methodology.

**Fig. 3:**
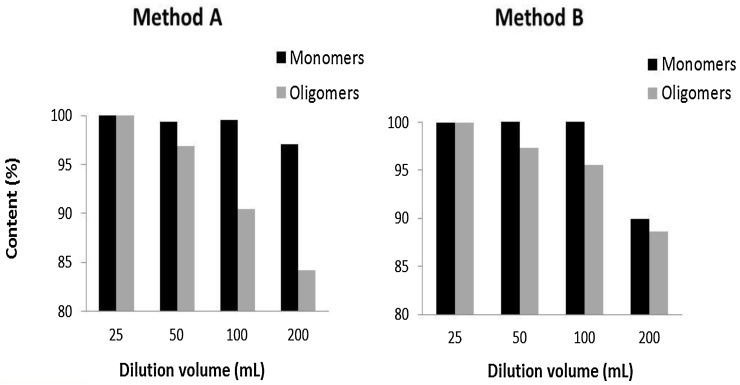
% reduction in monomeric and oligomeric procyanidin content of an apple extract by two dilution methods. n = 3 replicates per dilution volume. Oligomers are the summation of total procyanidins (dp2-10).

**Fig. 4:**
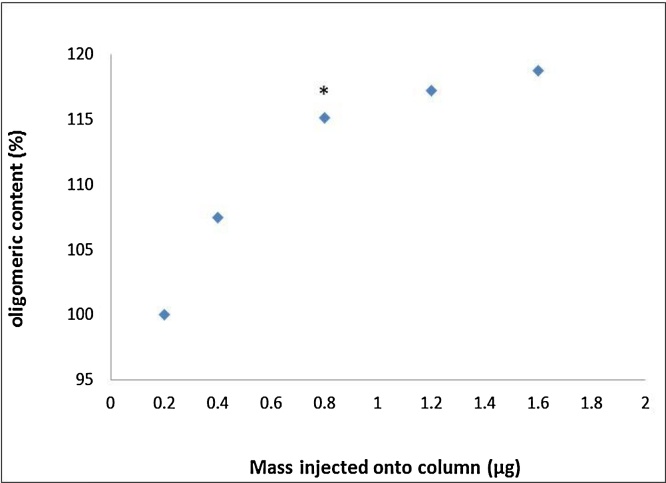
Oligomeric content (%) of an apple extract by mass injected onto the chromatographic column. Hilic column 150 × 2 mm; 3 μm internal diameter; 2uL injection volume * Equivalent to 100 mg apple extract in 50 mL 70% methanol.

Having determined an appropriate sample mass, we next investigated different dilution volumes to determine the optimal conc. of apple extract required for identifying each of the oligomeric peaks in a single chromatographic run. Our initial approach to dilute the apple extract was method A (Section 2.6.3). However, with this approach we observed a marked step-wise reduction in oligomeric procyanidin conc. of the extract as the final dilution volume increased (Fig. 3; **panel A**). Consequently, we tried a different approach to dilute the apple extracts (Method B, direct dissolution in final volume instead of step-wise dilution). Whilst we still observed an inverse correlation between the degree of dilution and oligomeric procyanidin conc., the effects were much less prominent compared with method A (Fig. 3; **panel B**). The expected procyanidin content of the material used for these experiments was ∼ 20%. On this basis, we calculated that injecting less than 0.8 μg oligomeric procyanidins onto the chromatographic column results in significant under quantification of these compounds in the apple extract (Fig. 4) using our method.

One likely explanation for these observations is adsorption of the procyanidins onto the surface of the glass volumetric flasks. Polar Interactions in flavanol adsorption onto solid surfaces have been reported previously [22]. Therefore, the inner surface area of the flasks used, were calculated. A significant correlation was observed between the concentrations and glass surface the solution had been in contact with. Cumulative surface area was used for the subsequently diluted solutions. Hereby, the slope for the subsequent dilutions was higher, confirming the hypothesis of glass wall adsorption. We repeated method B substituting glass with polypropylene volumetric flasks but the effects remained the same (data not shown). This effect cannot be overcome. Its impact can be reduced by using flasks of the same volume throughout the procedure.

### 3.2 Method validation

The performance characteristics as specified above were determined and the method was successfully validated. Results of the various experiments are described below.

#### 3.2.1 Linearity and working range

A calibration curve was constructed for epicatechin by random injection of standard solutions. The fluorescence detector response to epicatechin was plotted against a series of concentrations (5–100 μg/mL) and linearity determined visually and by means of a lack-of-fit-test. Forcing the calibration through zero was statistically supported as b is larger than the standard error of correlation (se_y_). The calculated F-value was smaller than the critical F-value for all congeners, indicating compliance with the linear model. To support the choice for forcing the calibration through zero, error for both approaches were calculated. By forcing the curve through zero, error at the lower end was significantly reduced.

The calibration curve for epicatechin was prepared over three separate days to assess linearity and a correlation coefficient of >0.999 achieved.

#### 3.2.2 Determination of RRFs

RRF for dp2-10 were determined from the epicatechin response factor and individual calibration curves for dp2-10 over the same range and on three separate days. The RRF for dp2-10 are listed in Table 2. Because of the nature of fluorescence detection and its sensitivity to the composition of the HPLC mobile phase solvents, these response factors are specific for the established method described in Section 2.7. The precision of RRF determination is further described in Section 3.2.4.

#### 3.2.3 LOQ

A theoretical LOQ was calculated at 0.5 μg/mL based on system performance and equation 1. Although lower LOQs were technically possible seeing the calibration through the origin was allowed, there was no need to do so for the samples under study. Therefore, the LOQ was set at the lowest point of the calibration points used to determine the RRFs. In practice, this resulted in the following LOQs: 5 μg/mL for epicatechin & dp2-5, 10 μg/mL for dp6, 25 μg/mL for dp7-9 and 50 μg/mL for dp10.

#### 3.2.4 Method precision

Table 3 summarises the precision of the analytical methodology. Method precision is expressed as the RSD between replicate measurements. Repeatability ranged from 2 to 6% depending on the analyte. Intermediate precision was good up to dp7 (ranging from 2 to 8%) and increasing to 10–13% for the higher dp’s. To assess the inter-day precision at the higher dp, participating laboratories of the inter-laboratory exercise were instructed to perform the analyses on different days.

**Fig. 5:**
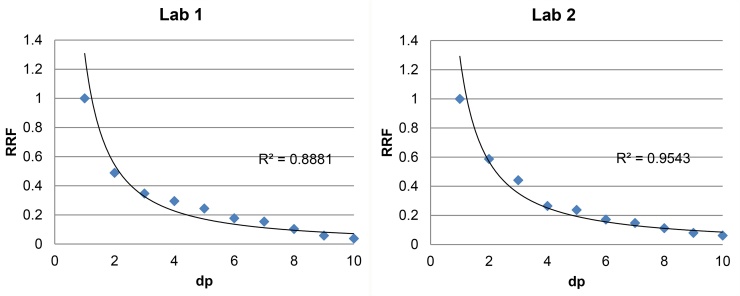
RRF correlation of different polymer chain lengths determined in 2 different laboratories.

Reproducibility of RRF determination was assessed. The RRF for the individual oligomers was determined at two locations, by different analysts, using different instrumentation. The RRFs for all analytes is given in Table 4. The correlation of the RRFs with the polymer chain length is visualised in Fig. 5. Variation within the RRFs of dp2-10 vs epicatechin adds to the method uncertainty and was considered in the final uncertainty estimation.

#### 3.2.5 Method trueness

Estimation of trueness was determined by fortifying an apple extract with known amounts of procyanidins as previously described. Trueness is expressed as% recovery of the analytes. Up to dp3 the trueness was >95%. However, trueness of the sample decreased as the dp increased.

The mean analyte concentrations obtained for the spiked samples (in μg/mg) were referred to the amount added to the samples of apple extracts in order to find an estimate of the response originated by the analytes spiked in the samples. The expected values were plotted against the concentrations that were actually found. An overview of the recoveries for all analytes is given in Table 5.

#### 3.2.6 Method uncertainty

The expanded method uncertainty lies between 12% and 103%, depending upon the analyte. Uncertainty increases with increasing degree of polymerisation. This is not unexpected as it is an indirect method that makes use of the RRFs to calculate results for higher dp’s. An overview of U for each dp is given in Table 6

#### 3.2.7 Stability of the extracts

Results of the stability experiments are summarized in Table 7. No trend could be observed and the extracts were considered stable within the studied timeframe. It is however advisable to complete the LC-measurements within one week from the moment of sample preparation to prevent drying of the extracts.

### 3.3 Inter-laboratory validation of the analytical method

Eight laboratories received the materials required to participate in the evaluation of the method for extracting and quantifying procyanidins from an apple extract. All laboratories were instructed to report all quantifiable analytes up to dp10. It was expected that all laboratories would be able to report procyanidins up to dp6 or 7 (extract A) and dp10 (extract B) using the methodology described. All eight laboratories returned results. Seven of the eight laboratories were able to measure and quantify all the expected analytes and returned full datasets. One laboratory returned a very limited dataset. The initial step in the evaluation of the results was to assess the quality of the returned data by means of the control sample. As a consequence of this assessment, one laboratory did not produce data in accordance with the desired quality (far outside confidence interval of consensus value) and was therefore not used for further evaluation. Seven full datasets were retained for evaluation. Results from the seven laboratories are presented in Table 8. The reported data are the mean results of six independent measurements over two days (extract A and B) and one independent measurement per day for the control sample. The values for the oligomeric procyanidins in Table 8 are the summation of all the reported individual analytes for each extract.

#### 3.3.1 Within-laboratory precision of the method

For monomeric procyanidins inter-day variance in measurement performance was exceptionally good, with six of the seven participants reporting% RSD values below 5% for both extracts and control sample. Inter-day variation in the measurement of total oligomeric content for each laboratory is also good, with most laboratories reporting RSD below 10%. On an individual analyte basis, measurement precision decreased with the higher dp’s which was not totally unexpected (data not shown).

#### 3.3.2 Between-laboratory precision of the method

For monomeric procyanidins, between laboratories precision of the method was very good, with reported RSD values ranging between 4 and 7% depending upon the extract (Table 8). Similarly, precision was good for oligomeric procyanidin quantification in extract A. For extract B, % RSD was somewhat higher. This was largely the consequence of one participant (number five) under-estimating total oligomer content quite significantly compared with the other six, despite identifying and quantifying all of the expected analytes in this material. Further scrutiny of the data revealed that the under-estimation was greater towards the higher end of the chromatogram (dp 4–5 upwards).

#### 3.3.3 Inter-laboratory performance

The quantitative criteria for the evaluation of the inter-laboratory performance, was determined using the z-score and calculated using the following equation:

$$Z-SCORE=\frac{X-\mu }{\sigma }$$

where x is the laboratory result; μ is the average of all participants and *σ* is the standard deviation of all results. The classification used to describe method performance is a z-score: ≤2 = satisfactory; <3 = doubtful; >3 = unsatisfactory.

All calculated z scores were below two (Table 9**)**, from which can be concluded that results were satisfactory. Close inspection of the z-scores and their statistical descriptors, and thus the labs’ performance, show that between lab bias is predominant over within lab variation. Average and median z-scores per lab are closely clustered together, which results in certain labs reporting on the high end for all analytes, while others are more bound to be on the lower end for all analytes. This bias however is within expectations as no z-score is bigger than two for this dataset. We can thus conclude that the method performance is under control.

### 3.4 Method performance compared to state-of-the-art

The method previously developed by Robbins et al. [15] has proven to be very useful, robust and has been applied to quantify cocoa procyanidins in a number of published studies. Nevertheless the currently developed and applied method proves to be superior in certain aspects. This method is significantly faster with the final detectable oligomer eluting around 38 min compared to 56 min for the Robbins method (a 30% improvement). In addition, whereas Robbins et al. [15] have validated for total content (sum of monomers and procyanidins), the present method provides performance characteristics for individual analytes. This is also the case for the total recovery, where Robbins et al. [15] reported around 93% for the entire test matrix, whereas here we have estimated an in-depth recovery of each individual oligomer. Further, our recovery estimates were averaged over different spiking levels which can be considered a more conservative approach. Nevertheless, re-calculation of the recovery as was reported by Robbins et al. [15] (i.e. 1 point spike vs non-spiked matrix), shows a total recovery of 95% for the sum of analytes, which is the same as reported previously by Robbins et al. Regarding precision and recovery, both methods appear to be comparable. However, it is important to note that the results of the current study have shown that using a generalised precision and recovery assessment (i.e. for the sum of analytes) is not ideal, as both validation parameters are compound dependent (at higher polymerisation degree both an increasing intermediate precision and decreasing recovery is observed). This justifies the conservative approach that was adopted in the current study, leading to what we consider a realistic measurement uncertainty.

The method reported by Robbins et al. [15]. was developed for cocoa powders and chocolate products, whereas the method described here was for apples and apple extracts. Since there are differences between apples and chocolate in terms of the matrices (the sugars present in the apple extract are of a different nature to those present in chocolate, there are likely to be differences in the nature of the procyanidins, the nature of the other non-flavan-3-ol polyphenols are different, and there are numerous non-phenolic compounds that are very different between the two materials), it was important to develop and validate a method specific for apple flavan-3-ols that allowed accurate quantification of individual oligomers. In summary, both methods perform equally well, with the one presented here outperforming that of Robins et al. [15]. in terms of speed and resolution. Further, the current method is more extensively validated and has been ascribed a measurement uncertainty.

## 4 Conclusion

An analytical method for the extraction, separation, identification and quantification of procyanidins in an apple extract was developed, validated and assessed. The established method utilizes a HILIC stationary phase with a binary mobile phase consisting of acidic acetonitrile and acidic aqueous methanol. The developed method was validated for standard linearity, RRF, sensitivity, method precision and trueness. Evaluation of the validated method was conducted by seven participating laboratories in seven countries across Europe. The results of the evaluation indicate that the established analytical method is reliable and reproducible. The method is the first reported for accurate quantification of individual procyanidins in apple extracts, and an improvement on an existing method for quantifying procyanidins in cocoa in terms of run time, resolution of analytes, the extent of validation and the inclusion of an estimate of measurement uncertainty.

## Conflict of interest

The authors declare no conflict of interest.

## Figures and Tables

**Table 1:** The effects of sample mass on variation in procyanidin conc. in an apple extract.

	% RSD per mass extract		
Analyte	20 mg	40 mg	100 mg
Monomer	13.9	1.5	0.6
dp2	14.5	4.6	0.7
dp3	24.9	5.7	1.4
dp4	9.4	3.9	1.2
dp5	36.3	5.3	1.6
dp6	36.2	5.8	1.9
dp7	43.1	5.2	0.7

Final dilution volume = 100 mL per mass of extract; RSD based on 3 replicates per mass of extract.

**Table 2:** Retention times and relative response factors for dp2-10.

Analyte	RT	Mean of means RRF	RSD RFF (%)
Epicatechin	2.1	1	4
dp2	4.8	0.587	4
dp3	12.0	0.442	5
dp4	15.9	0.265	12
dp5	18.7	0.238	10
dp6	21.0	0.172	9
dp7	23.6	0.148	9
dp8	26.5	0.113	19
dp9	29.5	0.080	34
dp10	32.4	0.062	4

n = 3 replicates per analyte.

**Table 3:** Method precision.

Analyte	Repeatability (intra-day)	Intermediate precision (inter-day)
Epicatechin	4%	2%
dp2	6%	2%
dp3	6%	3%
dp4	5%	2%
dp5	3%	4%
dp6	2%	7%
dp7	2%	8%
dp8	3%	10%
dp9	4%	13%
dp10	5%	13%

N = 3 replicates per analyte.

**Table 4:** Relative Response Factors (RRFs; mean-of-means) for the different analytes at the 2 test locations.

	Epicatechin	dp2	dp3	dp4	dp5	dp6	dp7	dp8	dp9	dp10
Lab 1	1	0.489	0.347	0.295	0.244	0.177	0.154	0.103	0.058	0.038
Lab 2	1	0.587	0.442	0.265	0.238	0.172	0.148	0.113	0.080	0.062

**Table 5:** Average recoveries and their estimated uncertainty based on spiking experiments.

	rec, %	urec, %
epicatechin	96%	7%
dp2	95%	8%
dp3	97%	6%
dp4	83%	7%
dp5	77%	7%
dp6	69%	11%
dp7	70%	9%
dp8	60%	11%
dp9	63%	15%
dp10	49%	34%

**Table 6:** Estimated expanded uncertainty.

Analyte	U_opt_, %	U_max_, %
epicatechin	12%	19%
dp2	17%	41%
dp3	16%	48%
dp4	42%	50%
dp5	51%	54%
dp6	65%	70%
dp7	64%	69%
dp8	88%	94%
dp9	101%	122%
dp10	103%	148%

**Table 7:** Stability assessment (mg/g analyte).

Compound	T0	48h	96h	168h	ip	Delta T0-168h	acceptable range (±)
Epicatechin	12.9	12.4	12.4	12.4	4%	−0.5	0.50
dp2	40.6	39.9	40.7	40.8	6%	0.2	2.43
dp3	45.1	45.4	45.9	45.7	6%	0.6	2.73
dp4	82.0	78.6	81.4	79.6	5%	−2.4	4.02
dp5	77.1	72.7	71.8	74.0	3%	−3.0	2.22
dp6	93.5	91.0	91.1	91.6	2%	−1.9	1.84
dp7	78.6	81.9	77.7	73.3	2%	−5.3	1.56
dp8	71.5	69.7	66.7	67.9	3%	−3.6	2.07
dp9	61.6	61.5	64.7	64.4	4%	2.8	2.52
dp10	54.9	52.2	52.5	53.2	5%	−1.7	2.66

**Table 8:** Monomeric and total oligomeric procyanidin conc. (mg/g) determined by seven laboratories.

	Extract A				Extract B				Control			
Lab	Monomers		Oligomers		Monomers		Oligomers		Monomers		Oligomers	
1	337.3	(3.4)	324.5	(2.5)	14.3	(2.7)	495.5	(2.0)	2.9	(2.7)	8.0	(2.6)
2	344.2	(0.9)	381.6	(2.4)	12.5	(3.0)	601.7	(1.0)	2.8	(0.9)	10.7	(10.0)
3	323.8	(2.1)	317.9	(1.8)	12.3	(4.9)	445.7	(6.3)	2.7	(9.0)	7.4	(5.6)
4	332.0	(13.9)	296.6	(12.2)	13.1	(14.8)	439.5	(9.7)	3.1	(1.4)	4.7	(17.6)
5	323.2	(2.3)	233.4	(2.7)	12.5	(3.0)	187.9	(4.8)	3.0	(0.7)	5.7	(6.9)
6	306.2	(0.1)	275.4	(0.1)	12.6	(0.3)	373.0	(2.2)	3.0	(0.6)	7.5	(1.3)
7	312.0	(0.8)	277.1	(0.7)	11.4	(1.6)	408.8	(5.4)	2.6	(1.9)	4.5	(12.1)
Mean	325.5		300.9		12.7		421.7		2.9		6.9	
SD	13.5		46.8		0.9		126.4		0.2		2.2	
% RSD	4.1		15.6		7.0		30.0		6.6		31.6	

Mean (RSD) of six independent measurements of two apple extracts analysed on two different days (i.e. n = 3 replicates/extract/day) and a control sample (n = 1/day).

**Table 9:** Z-scores of all analytes per participant for both test samples.

Lab		1	2	3	4	5	6	7
Extract A	epicatechin	−0.10	−1.46	0.86	0.47	−0.14	−1.00	1.37
	dp2	−1.19	−1.10	0.38	0.63	0.52	−0.68	1.45
	dp3	−1.47	−0.53	0.51	−0.24	0.81	−0.57	1.50
	dp4	−1.50	−0.31	0.63	−0.29	−0.24	−0.05	1.76
	dp5	−1.44	−0.05	0.67	−0.83	−0.27	0.31	1.62
	avg	−1.14	−0.50	0.55	−0.18	0.21	−0.25	1.58
	med	−1.44	−0.42	0.57	−0.26	0.14	−0.31	1.56
	min	−1.50	−1.46	0.38	−0.83	−0.27	−1.00	1.37
	max	−0.10	−0.05	0.86	0.63	0.81	0.31	1.76

Extract B	epicatechin	−0.19	−0.13	1.85	0.50	−0.40	−1.45	−0.18
	dp2	−1.62	−0.63	0.08	0.90	0.72	−0.60	1.14
	dp3	−1.43	−0.97	0.65	0.44	0.41	−0.50	1.40
	dp4	−1.59	−0.75	0.64	0.42	0.00	−0.23	1.51
	dp5	−1.92	−0.52	0.55	0.63	0.15	−0.03	1.15
	dp6	−1.96	−0.30	0.41	0.49	−0.02	0.09	1.29
	dp7	−1.92	−0.13	0.46	−0.11	0.01	0.27	1.41
	dp8	−1.87	−0.19	0.49	−0.34	0.18	0.32	1.41
	dp9	−1.86	−0.38	0.59	−0.17	0.26	0.18	1.38
	dp10	−1.69	−0.21	0.75	−0.80	0.27	0.39	1.29
	avg	−1.61	−0.42	0.65	0.20	0.16	−0.16	1.18
	med	−1.78	−0.34	0.57	0.43	0.16	0.03	1.33
	min	−1.96	−0.97	0.08	−0.80	−0.40	−1.45	−0.18
	max	−0.19	−0.13	1.85	0.90	0.72	0.39	1.51
